# Videos in short-video sharing platforms as sources of information on osteoarthritis: cross-sectional content analysis study

**DOI:** 10.3389/fdgth.2025.1622503

**Published:** 2025-09-05

**Authors:** Yi-xiang Zhang, Hao-tian Yin, Ya-xing Liu, Xin Fu, Jun Liu

**Affiliations:** ^1^ Department of Joints, Tianjin Hospital of Tianjin University (Tianjin Hospital), Tianjin, China; ^2^ Clinical College of Orthopedics, Tianjin Medical University, Tianjin, China; ^3^ Tianjin University, Tianjin, China

**Keywords:** osteoarthritis, short videos, health information, quality assessment, reliability

## Abstract

**Background:**

Osteoarthritis (OA) is a debilitating condition characterized by pain, stiffness, and impaired mobility, significantly affecting patients' quality of life. Health education is crucial in helping individuals understand OA and its management. In China, where OA is highly prevalent, platforms such as TikTok, WeChat, and XiaoHongshu have become prominent sources of health information. However, there is a lack of research regarding the reliability and educational quality of OA-related content on these platforms.

**Methods:**

This study analyzed the top 100 OA-related videos across three major platforms: TikTok, WeChat, and XiaoHongshu. We systematically evaluated the content quality, reliability, and educational value using established tools, such as the DISCERN scale, JAMA benchmark criteria, and the Global Quality Score (GQS) system. The study also compared differences in video content across platforms, offering insights into their relevance for addressing professional needs.

**Results:**

Video quality varied significantly between platforms. TikTok outperformed WeChat and XiaoHongshu in all scoring criteria, with mean DISCERN scores of 32.42 (SD 0.37), 24.57 (SD 0.34), and 30.21 (SD 0.10), respectively (*P* < 0.001). TikTok also scored higher on the JAMA (1.36, SD 0.07) and GQS (2.46, SD 0.08) scales (*P* < 0.001). Videos created by healthcare professionals scored higher than those created by non-professionals (*P* < 0.001). Disease education and symptom self-examination content were more engaging, whereas rehabilitation videos received less attention.

**Conclusions:**

Short-video platforms have great potential for chronic disease health education, with the caveat that the quality of the videos currently varies, and the authenticity of the video content is yet to be verified. While professional doctors play a crucial role in ensuring the quality and authenticity of video content, viewers should approach it with a critical mindset. Even without medical expertise, viewers should be encouraged to question the information and consult multiple sources.

## Introduction

Osteoarthritis (OA) is a chronic degenerative joint disease characterized by progressive wear and degeneration of articular cartilage, often accompanied by damage to the surrounding soft tissues (1–3). It is recognized as a major cause of disability and motor dysfunction worldwide. Data from the World Health Organization show that the global prevalence of OA is approximately 5%, and its incidence increases with age (1, 4). OA causes not only persistent joint pain, limitation of movement, and morning stiffness, but also joint swelling. In advanced stages, OA may result in joint deformity and even disability, significantly impairing patients' ability to perform daily activities and affecting their quality of life (3). Although OA remains incurable, comprehensive interventions—including standardized pharmacological treatment, physical therapy, injection therapy, exercise rehabilitation, and surgery when necessary can effectively alleviate symptoms and significantly improve patients' quality of life (1, 2, 5–7).

Health education plays a pivotal role in the management of OA by equipping patients with the knowledge necessary to manage this chronic condition. Enhancing public awareness facilitates a better understanding of OA pathogenesis, common symptoms, and available treatment options (8, 9), thereby promoting early detection, timely intervention, and appropriate management. The rapid development of information technology and the proliferation of short video platforms have led to transformative changes in the form of health education. Short video, as an emerging form of social media, have quickly become a major vehicle for health science dissemination (10–13). Platforms such as TikTok, WeChat Video Accounts, and Xiaohongshu have transcended their purely entertainment and social functions, becoming essential platforms for health education among Chinese people (14–16).

A significant amount of OA-related content has emerged on short-form video platforms, ranging from specialized medical information to personal patient experiences and discussions of various treatment options. A major issue is that creators possess varying levels of professionalism, resulting in mixed content quality and raising concerns about the accuracy, reliability, and educational value of the videos. Studies have shown that many medical science videos lack a solid scientific foundation, and numerous content creators disseminate misleading information in pursuit of increased traffic and viewer engagement (17, 18). For instance, some so-called “special effect treatments” are not only ineffective but may also delay proper treatment. Therefore, it is crucial to conduct a comprehensive evaluation of OA-related videos on these platforms to rigorously assess their quality and credibility. This is not only a critical step in enhancing the effectiveness of public health education, but also a necessary measure to ensure the public has access to accurate and reliable health information.

In recent years, researchers have frequently utilized specialized tools, such as the DISCERN scale, the JAMA criteria, and the Global Quality Score (GQS), to assess three key dimensions: reliability, completeness, and medical standardization of video content (19–25). D’Ambrosi et al., analyzed 100 videos related to humeral epicondylitis on the Shakeology platform (26). The study found that the videos were posted by physical therapists and focused on rehabilitation exercises. However, despite the high number of clicks and interactions, the quality and credibility of the videos were questionable. Other similar studies have highlighted a paradox: short video platforms can quickly disseminate health information, yet improving the quality of content remains a persistent challenge (27–29).

Previous studies have primarily focused on health-related videos on platforms such as YouTube and Instagram, with limited research conducted on Chinese short-video platforms. OA, a highly prevalent and disabling condition, has not yet been systematically assessed in terms of the quality of related health content, despite its undeniable importance. This study aims to critically assess the top 100 OA-related videos on Chinese short-video platforms such as TikTok, WeChat, and Xiaohongshu, using tools like DISCERN, the JAMA benchmark, and GQS to evaluate their quality, reliability, and educational value, thereby addressing gaps in previous research. Additionally, this study explores the impact of short-video platform algorithms on the visibility and quality of OA-related content, revealing how algorithm-driven recommendation mechanisms shape health education in the digital age.

## Methods

### Ethical considerations

This study focused solely on data obtained from publicly accessible short-video sharing platforms, with no involvement of human experimental research, thereby obviating the need for ethical approval. Furthermore, no identifiable information regarding individual users or their IDs was included in this study, ensuring privacy and confidentiality.

### Search strategy and data collection

In designing our search and data collection strategy, we referred to established methodological guidelines for conducting health research using data from social media platforms, which provide a step-by-step framework for healthcare professionals and researchers to follow (30). A search was performed on TikTok, WeChat, and Xiaohongshu from March 1 to March 6, 2025, using the keyword “骨关节炎” (osteoarthritis). To minimize bias from personalized recommendation algorithms, searches were performed while logged out and using newly created accounts. The search results from each platform were sorted according to the default ranking algorithms, without applying any preset filters (e.g., relevance, most viewed). This approach ensured that the collected videos adhered to the platform's standard sorting criteria. We selected the top 100 videos for each search term to ensure a representative sample of the most relevant content (31). Inclusion criteria were as follows: (1) videos related to OA, and (2) videos in Chinese. Exclusion criteria were: (1) duplicate content, (2) advertisements, and (3) videos containing irrelevant content. For the selected videos, characteristics such as titles, number of likes, comments, collections, shares, video duration, sources, type of information, and content were recorded and analyzed.

### Primary and secondary outcomes

The primary outcomes of this study were the reliability and quality of OA-related videos, evaluated using the DISCERN scale, JAMA benchmarks, and the GQS. These outcomes aimed to assess the accuracy and overall quality of the health information presented in the videos. The secondary outcomes included engagement metrics (likes, comments, shares, collections). The understandability of the videos was also assessed using the Patient Education Materials Assessment Tool (PEMAT).

### Exploratory analyses

In addition to the primary and secondary outcomes, we conducted exploratory analyses to examine differences in video quality based on the content creator type. Specifically, we compared the DISCERN, JAMA, and GQS scores for videos created by healthcare professionals (e.g., physicians, rehabilitation practitioners) and independent users. Furthermore, we examined how the content type influenced engagement metrics (likes, comments, shares, collections). This study also analyzes the impact of platform algorithms on content visibility and recommendation quality through correlation analysis. Specifically, it explores how algorithm-driven factors, such as user engagement (likes, comments, shares), correlate with video quality scores (e.g., DISCERN, JAMA, GQS).

### Assessment tools for video reliability, validity, and quality

#### DISCERN instrument

The DISCERN tool is a structured assessment scale designed to evaluate the reliability and quality of information for both patients and healthcare providers. Items 1–8 form the first section, focusing on evaluating the reliability of the information. Items 9–15 form the second section, evaluating the quality of the information, while the final item (16) provides an overall quality rating. The DISCERN tool uses a five-point Likert scale for evaluation. For the first 15 items, a score of 1 indicates “no,” while a score of 5 indicates “yes.” For item 16, a score of 1 signifies “low quality with significant deficiencies,” while a score of 5 signifies “high quality with minimal deficiencies.” The total DISCERN score is calculated as the sum of the first 15 items, ranging from a minimum of 15 to a maximum of 75. A higher score indicates greater reliability and quality of the information—scores of 15–27 indicate “very poor,” 28–38 indicate “poor,” 39–50 indicate “medium,” 51–62 indicate “good,” and 63–75 indicate “excellent.” The DISCERN tool is freely accessible at http://www.discern.org.uk (19, 23).

#### JAMA benchmark criteria

The JAMA benchmark criteria tool is is one of the most widely used tools for assessing medical information sourced from online platforms. It comprises four criteria: authorship, attribution, disclosure, and currency, each with a maximum score of one point, resulting in a total possible score of 4 points. In the JAMA evaluation, a score of 0–1 points indicates insufficient information, 2–3 points indicates partially sufficient information, and 4 points indicates completely sufficient information (24, 25).

#### Global quality score

The GQS is a scoring system developed to evaluate the instructional quality of videos. It enables the assessment of the quality, accessibility, and usability of information in online videos. In the GQS evaluation, a score of 1 indicates the lowest quality and limited usefulness for viewers, while a score of 5 indicates excellent quality and substantial usefulness (20, 21).

To further assess the understandability and actionability of the videos, the PEMAT, developed by the Agency for Healthcare Research and Quality, was employed (32). To minimize potential bias, all data collection and statistical analyses were conducted by a designated tester throughout the trial. Two raters, both senior clinicians with over 10 years of experience, independently evaluate the content of each video. They discuss and resolve any disagreement. In cases where consensus cannot be reached—a third rater, an experienced department head with extensive clinical expertise—will intervene to provide the final score.

### Statistical analysis

Descriptive statistics were used to analyze all video features, including video source, content, audio, and message type (i.e., DISCERN, JAMA, and GQS). Categorical variables were presented as absolute frequencies and percentages, while continuous variables were reported as mean ± standard deviation, or alternatively as median, interquartile range (IQR), and range. Normally distributed data were presented as mean ± SD, while non-normally distributed data were reported as median and range. Comparisons between two groups were conducted using the Mann–Whitney *U* test or the Student's *t*-test, while comparisons among three or more groups were performed using the Kruskal–Wallis test or one-way ANOVA. Spearman correlation analysis was used to assess the relationship between quantitative variables. *P* value less than 0.05 (*P* < 0.05) was considered statistically significant. All statistical analyses were conducted using SPSS (version 22.0; IBM Corp) and GraphPad Prism (version 9.0; Dotmatics).

## Results

### The general characteristics of videos

**Figure 1:**
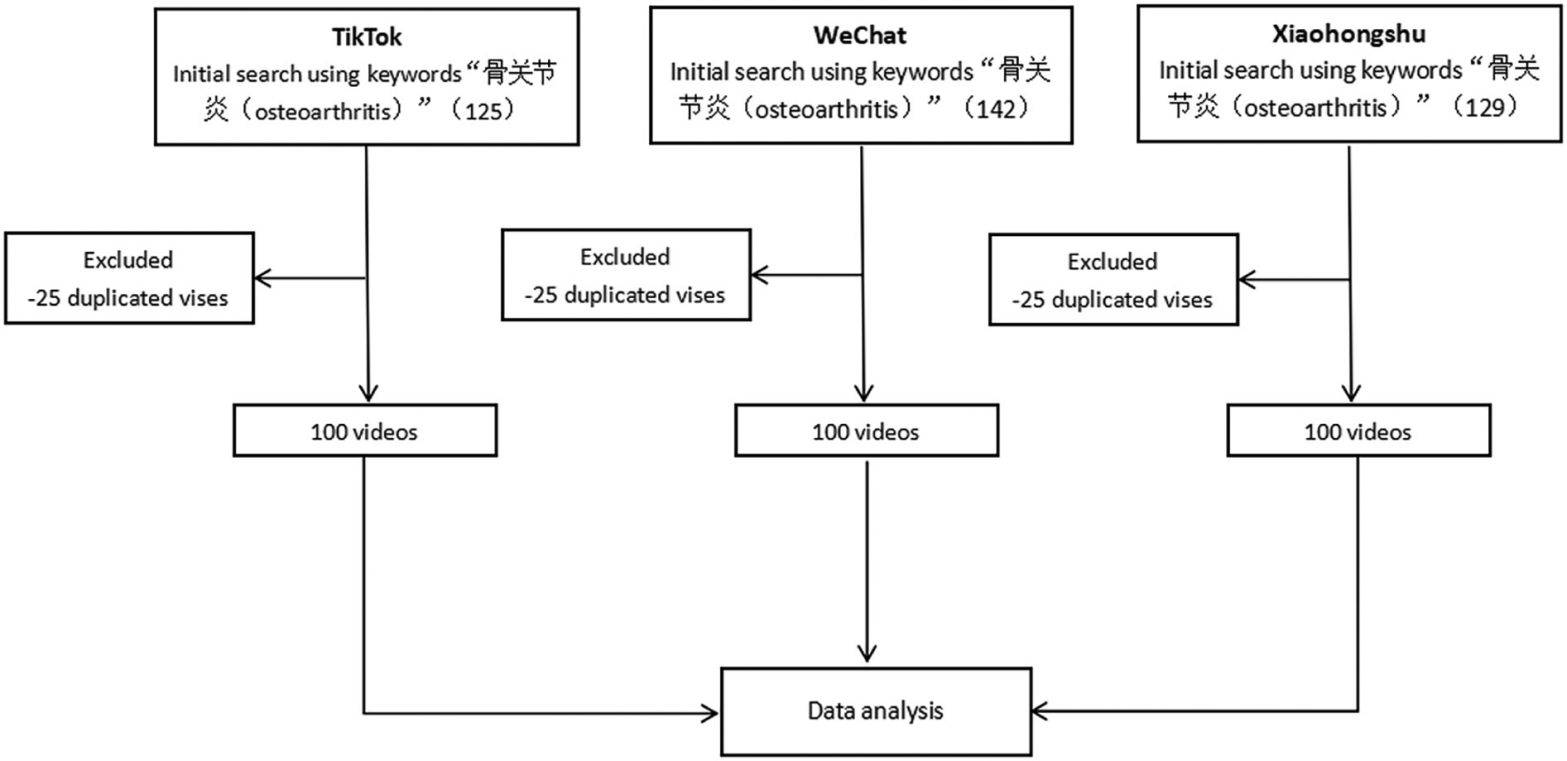
Flowchart of the study.

As shown in Figure 1, TikTok, WeChat, and Xiaohongshu each contained 100 videos after excluding advertisements, duplicates, and irrelevant content. Analysis of the general characteristics of the videos revealed no significant difference in video duration across the three platforms (*p* = 0.312). Videos on TikTok received more likes, comments, shares, and favorites than those from WeChat and Xiaohongshu (both *p* < 0.001). DISCERN ratings showed that TikTok videos scored higher than Xiaohongshu, which in turn scored higher than WeChat (mean 32.42, SD 0.37 vs. mean 30.21, SD 0.10 vs. mean 24.57, SD 0.34; *P* < 0.001); The JAMA score for TikTok videos was higher than that for Xiaohongshu, which was higher than that for WeChat (mean 1.36, SD 0.07 vs. mean 1.21, SD 0.05 vs. mean 0.76, SD 0.06; *P* < 0.001); The GQS score for TikTok videos was higher than for WeChat, which was higher than for Xiaohongshu (mean 2.46, SD 0.08 vs. mean 2.16, SD 0.06 vs. mean 1.90, SD 0.04; *P* < 0.001). Statistical differences were observed in the understandability and actionability of videos across different platforms (*P* < 0.001; Table 1). The detailed characteristics of OA-related videos across platforms are presented in Table 1.

**Table 1:** The general characteristics and scores of the osteoarthritis–related videos.

Parameters	TikTok (*n* = 100)	WeChat (*n* = 100)	Xiaohongshu (*n* = 100)	*P* value
Duration (seconds): median (range)	65 (7–182)	71 (27–900)	68 (11–216)	0.312
Likes: median (range)	878 (19–254,000)	68 (1–36,000)	14 (0–819)	<0.001
Comments: median (range)	38 (1–6,564)	4 (0–1,950)	1 (0–108)	<0.001
Shares: median (range)	144 (1–42,000)	89 (1–100,000)	5 (0–951)	<0.001
Collections: median (range)	278 (4–63,000)	51 (1–35,000)	8.5 (0–1,295)	<0.001
DISCERN score: median (range)	32.42 (0.37)	24.57 (0.34)	30.21 (0.10)	<0.001
JAMA^a^ score: mean (SD)	1.36 (0.07)	0.76 (0.06)	1.21 (0.05)	<0.001
GQS^b^ score: mean (SD)	2.46 (0.08)	2.16 (0.06)	1.90 (0.04)	<0.001
Understandability: mean (SD)	7.23 (0.13)	7.05 (0.19)	4.50 (0.10)	<0.001
Actionability: mean (SD)	6.19 (0.11)	6.50 (0.25)	5.21 (0.22)	<0.001

^a^ JAMA: Journal of American Medical Association.

^b^ GQS: global quality scale.

### Video source and content

Table 2 presents the sources and content of OA-related videos. Doctors were the primary uploaders of videos (255/300, 85.0%), and the content primarily focused on physical therapy (184/300, 61.3%) and etiology (41/300, 13.6%). The video content predominantly focused on educational science (215/300, 71.7%) and rehabilitation guidance (28/300, 9.3%).

**Table 2:** The sources and content of the osteoarthritis–related videos.

Variable		Total	TikTok, *n* (%)	WeChat, *n* (%)	Xiaohongshu, *n* (%)
Video source	Doctor	255	90 (90%)	81 (81%)	84 (84%)
	Physiotherapist	29	7 (7%)	11 (11%)	11 (11%)
	Private user	16	3 (3%)	8 (8%)	5 (5%)
Type of information	Physical therapy	185	56 (56%)	59 (59%)	70 (70%)
	Anatomy	23	10 (10%)	5 (5%)	8 (8%)
	Clinical examination	25	11 (11%)	7 (7%)	7 (7%)
	Etiopathogenesis	41	16 (16%)	16 (16%)	9 (9%)
	Patient experience	24	7 (7%)	13 (13%)	4 (4%)
Video content	Education	209	65 (65%)	69 (69%)	75 (75%)
	Rehabilitation	28	12 (12%)	8 (8%)	8 (8%)
	Clinical technology Demonstration	21	10 (10%)	4 (4%)	7 (7%)
	Patient experience/testimony	22	4 (4%)	11 (11%)	7 (7%)
	Research progress	14	9 (9%)	2 (2%)	3 (3%)

### The quality and popularity of videos from different sources with different contents and different presentation forms

**Figure 2:**
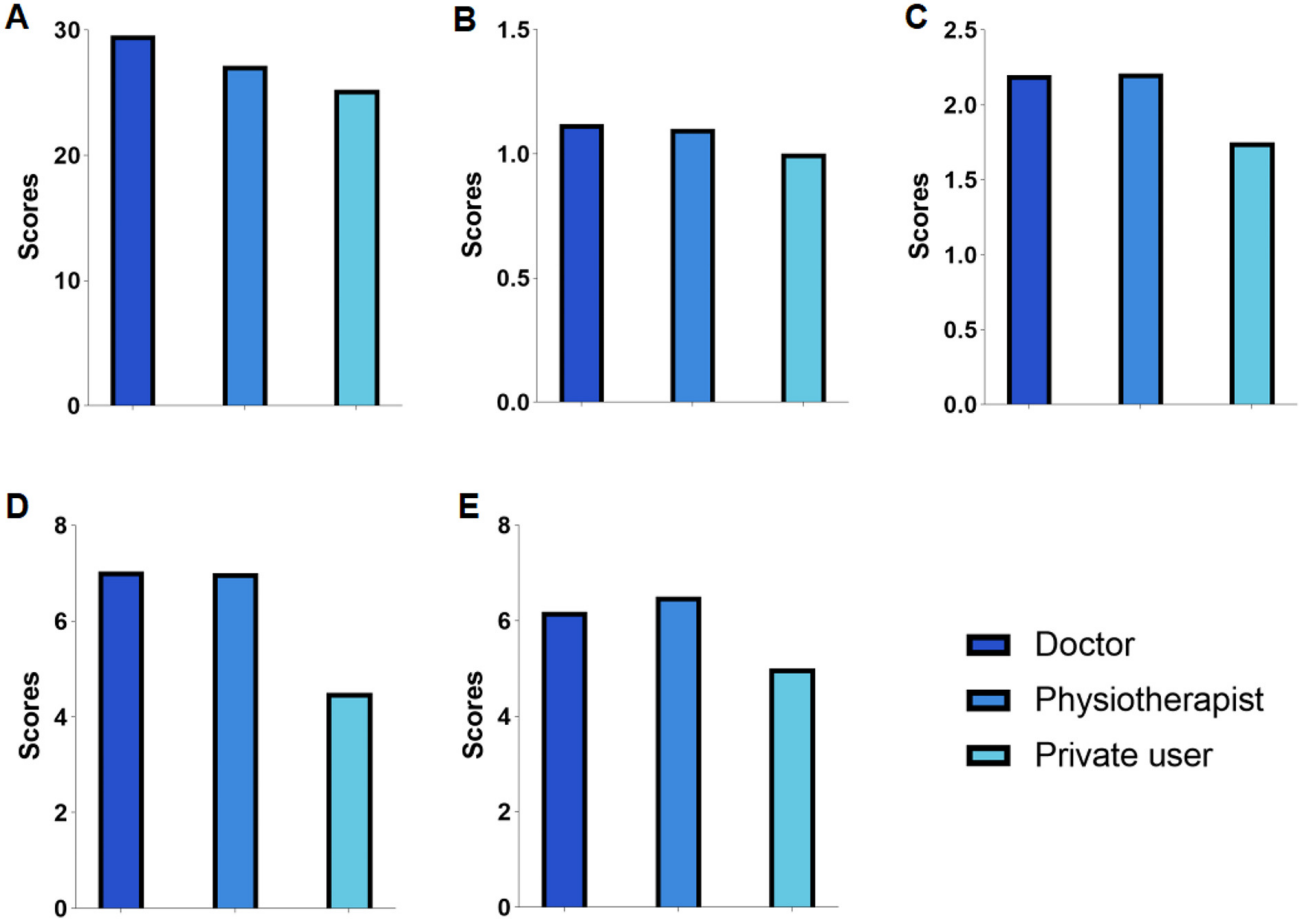
The journal of American Medical Association (JAMA) score, global quality scale (GQS) score, modified DISCERN score, patient education, materials assessment tool (PEMAT)–understandability, and PEMAT-actionability of videos on colorectal polyps from different sources. **(A)** The JAMA score, **(B)** the GQS score, **(C)** the modified DISCERN score, **(D)** PEMAT-understandability, and **(E)** PEMAT-actionability.

**Figure 3:**
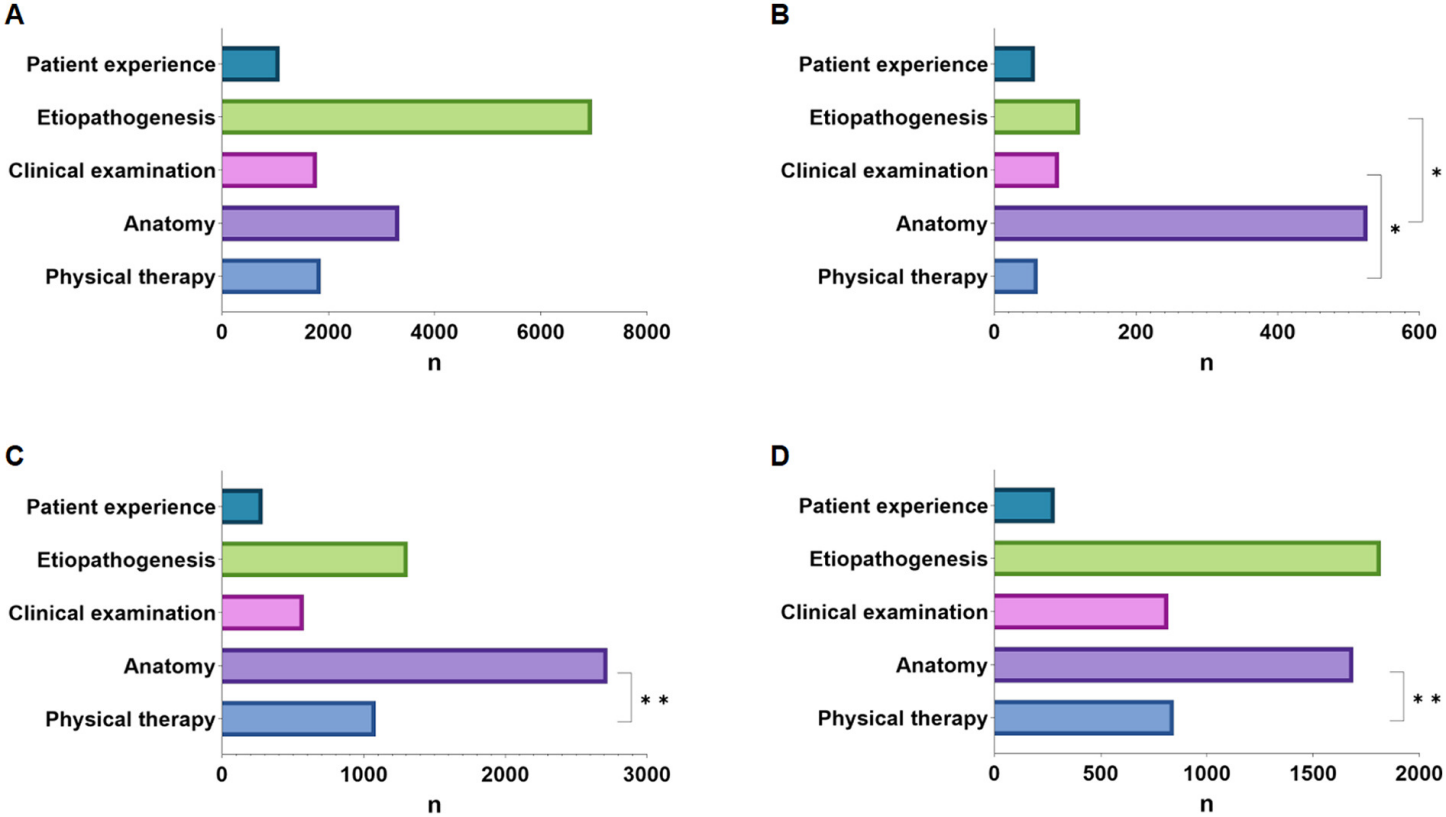
**(A)** The number of likes. **(B)** The number of discussions. **(C)** The number of shares. **(D)** The number of collections. (*P < .05, **P < .01).

When examining differences in information publishers, we found that videos uploaded by independent users had significantly lower DISCERN, JAMA, and GQS scores compared to those uploaded by physicians and rehabilitation practitioners, with significant differences observed (all *P* < 0.001; Table 3; Figures 2A–C). Similarly, the understandability and actionability of videos uploaded by private users were significantly lower than those uploaded by rehabilitators and physicians (*P* < 0.05, *P* < 0.001; Table 3; Figures 2D,E). When examining differences in the type of information, our study found no significant difference in likes across types (*p* = 0.053). However, regarding the number of discussions, clinical examination outperformed physical therapy (mean 91.12, SD 156.53 vs. mean 61.55, SD 216.80; *P* < 0.05), and anatomy outperformed etiopathogenesis (mean 526.65, SD 1438.78 vs. mean 120.40, SD 565.86; *P* < 0.05). Anatomy outperforms physical therapy in terms of shares (mean 2,720.85, SD 8,060.86 vs. mean 1,081.93, SD 7,717.04; *P* < 0.01). Patient experience ranked lower than anatomy in terms of number of collections (mean 284.08, SD 899.09 vs. mean 1,689.88, SD 3,875.34; *P* < 0.05) (Figures 3A–D).

**Table 3:** The popularity of videos from different sources.

Variable		Doctor *N* = 255 Mean ± SD	Physiotherapist *N* = 29 Mean ± SD	Private user *N* = 16	*P* value
Video score	DISCERN	29.56 ± 3.94	27.14 ± 5.62	25.25 ± 6.57	<0.001
	JAMA	1.12 ± 0.63	1.10 ± 0.67	1.00 ± 0.82	<0.001
	GQS	2.20 ± 0.67	2.21 ± 0.77	1.75 ± 0.77	<0.001
	Understandability	7.04 ± 0.56	7.00 ± 0.38	4.50 ± 0.70	<0.05
	Actionability	6.19 ± 0.57	6.50 ± 0.70	5.00 ± 0.62	<0.001

### Correlation analysis

Spearman correlation (*ρ*) analyses identified the relationships between various video variables. The results showed a positive correlation between video duration and video sharing (*P* < 0.05), but no correlation between duration and the number of likes, comments, or collections (all *P* > 0.05). And there is a significant positive correlation between the number of likes and the number of comments, shares and collections (*P* < 0.001; Table 4). The number of likes and comments were positively correlated with both the DISCERN and GQS scores (*P* < 0.001). The number of shares was positively correlated with the GQS score (*P* < 0.001). The number of collections was positively correlated with both the DISCERN and GQS scores (*P* < 0.01, *P* < 0.001; Table 5).

**Table 4:** The correlation analysis between the video variables.

Variables		Duration	Likes	Comments	Shares	Collections
Duration	*ρ*	1	0.097	0.028	0.143	0.109
	*P* value	–	0.094	0.633	0.013	0.061
Likes	*ρ*	0.097	1	0.894	0.866	0.929
	*P* value	0.094	–	<0.001	<0.001	<0.001
Comments	*ρ*	0.028	0.894	1	0.792	0.868
	*P* value	0.633	<0.001	–	<0.001	<0.001
Shares	*Ρ*	0.143	0.866	0.792	1	0.919
	*P* value	0.013	<0.001	<0.001	–	<0.001
Comments	*ρ*	0.028	0.894	1	0.792	0.868
	*P* value	0.633	<0.001	–	<0.001	<0.001
Shares	*ρ*	0.143	0.866	0.792	1	0.919
	*P* value	0.013	<0.001	<0.001	–	<0.001
Collections	*ρ*	0.109	0.929	0.868	0.919	1
	*P* value	0.061	<0.001	<0.001	<0.001	–

**Table 5:** The correlation analysis between video variables and the video quality.

Variables		DISCERN score	JAMA score	GQS score	Understandability	Actionability
Duration	*ρ*	−0.040	0.070	0.060	−0.104	0.196
	*P* value	0.489	0.229	0.298	0.453	0.307
Likes	ρ	0.286	0.125	0.363	0.029	−0.203
	*P* value	<0.001	0.031	<0.001	0.834	0.282
Comments	*ρ*	0.267	0.109	0.313	0.033	−0.191
	*P* value	<0.001	0.059	<0.001	0.812	0.312
Shares	*ρ*	0.150	−0.023	0.318	0.069	−0.097
	*P* value	0.307	0.694	<0.001	0.618	0.609
Collections	*ρ*	0.158	0.050	0.332	0.104	−0.082
	*P* value	0.006	0.393	<0.001	0.451	0.666

### Platform algorithm impact on video visibility and quality scores

Correlation analysis revealed a significant relationship between interaction metrics and video quality scores. Specifically, videos with higher interaction rates are generally associated with higher quality scores (Table 5). On TikTok, videos with high interaction rates and quality scores are significantly more prevalent than on other platforms (*P* < 0.001; Table 1), possibly due to TikTok's recommendation algorithm. TikTok's algorithm tends to prioritize videos with high interaction rates, which generally receive higher quality scores. After viewing high-quality videos, viewer interaction frequency tends to increase, creating a positive feedback loop. This finding further supports the potential influence of platform recommendation algorithms on video visibility and the quality of recommended content.

## Discussion

This study aims to assess the quality, reliability, and educational value of OA-related videos on Chinese short-video platforms, including TikTok, WeChat, and Xiaohongshu, thereby addressing a gap in existing research. The results indicate significant differences in video quality across these platforms, reflecting a broader trend in the dissemination of health education content via social media. The study also highlights the impact of short-video platform algorithms on video visibility and recommended content.

### Significance of the findings

The evaluation using the DISCERN, JAMA, and GQS revealed that OA-related videos on TikTok significantly outperformed those on Xiaohongshu and WeChat across multiple indicators. Specifically, TikTok videos were not only more credible but also more comprehensive and practical. This finding aligns with previous studies and confirms TikTok's leading role in health science popularization (33–35). This difference also highlights the potential and significance of TikTok in public health education, offering insight into how communication media directly influence the quality of health information.

The DISCERN and JAMA assessments revealed that videos created by healthcare professionals (e.g., physicians, rehabilitation therapists) received higher ratings compared to those produced by independent users. Videos created by non-professionals typically exhibited two major shortcomings: unclear presentation of specialized knowledge and a lack of practical instructional value, often aimed more at garnering attention than providing viewer benefit. These findings are consistent with previous studies (36, 37). These results emphasize that professional qualifications are crucial for ensuring the accuracy and credibility of health information, and that increasing the involvement of medical professionals in health education is essential for disseminating both professional and practical health knowledge to the public.

This study also found that content, subject matter, and presentation style significantly influenced user engagement (likes, shares, comments). Videos providing detailed explanations of bone and joint anatomy and clinical symptoms received more engagement than those focused on rehabilitation. Viewers preferred scientific content that was information-dense, detailed, and practically applicable, offering immediate utility. These findings are consistent with previous studies (38–40).

### Comparison with related literature

The results of this study demonstrate a high degree of consistency with previous research on social media health education. D’Ambrosi et al. found that, although physical therapists garnered significant attention on TikTok, the quality of their content varied (41). Similarly, several studies have emphasized the potential of short-form video platforms in advancing public health education (42, 43); however, the issue of fluctuating content quality persists, failing to persuade all viewers. These findings highlight a central paradox: when health discussions are dominated by laypeople, ensuring the accuracy and reliability of information becomes challenging. Our results are consistent with studies by Ozsoy (39) and Uprak (37) on YouTube health videos, which demonstrated that content created by healthcare professionals consistently outperformed that created by laypeople in terms of reliability and quality (37, 39). This finding achieves consensus across platforms and geographies, as an increasing number of researchers focus on online health issues. The deep involvement of healthcare professionals is indispensable for ensuring that video content is both credible and beneficial.

### Role of recommendation algorithms

Although recommendation algorithms significantly influence the dissemination of health information on short-video platforms, there has been limited research on the impact of platform-specific recommendation systems on the visibility and quality of health-related videos, particularly those concerning OA. This study is the first to examine how the recommendation algorithms of TikTok, WeChat, and Xiaohongshu influence the display, visibility, and dissemination effectiveness of video content. Using TikTok as an example, its algorithm primarily relies on user interaction metrics such as likes, comments, and shares to determine video recommendations (44–46). The results of this study suggest that videos with higher interaction metrics are generally associated with higher video quality. This positive feedback loop leads TikTok's algorithm to prioritize higher-quality OA-related videos, which subsequently generate more interaction. This interaction-based recommendation mechanism may cause certain health content to gain higher visibility, while content with fewer interactions may be overlooked, despite potentially possessing significant educational value. Similarly, WeChat and Xiaohongshu have their own recommendation algorithms, although their priorities may not rely solely on interaction data but also include content relevance and user behavior. Due to the differing recommendation mechanisms across these platforms, health videos about OA may display varying levels of visibility and dissemination patterns, thereby affecting the accuracy and breadth of health information accessible to users. This disparity highlights potential biases inherent in recommendation algorithms in health information dissemination. We believe that while platform recommendation algorithms can increase video exposure, they may inadvertently enhance the visibility of certain content, causing misleading videos to attract more audience attention. Consequently, algorithmic bias may have detrimental effects on the health education functions of these platforms (47, 48).

## Limitations and future directions

While this study offers valuable insights into the quality of OA-related videos on Chinese short-video platforms, several limitations and opportunities for future research remain.

### Artificial intelligence-based analysis

This study relied on manual scoring and conventional evaluation tools. Future research could leverage artificial intelligence (AI) and machine learning (ML) techniques to automate the classification and analysis of health-related videos (49). For instance, AI could be employed for content categorization, misinformation detection, and sentiment analysis, enabling large-scale analysis of videos and their potential impact on public health. Additionally, natural language processing (NLP) could be applied to analyze viewer comments and interactions, offering deeper insights into audience engagement, trust, and behavior (50).

### Expansion of data set and cross-platform/multilingual comparison

This study focused on a limited set of videos from Chinese platforms. Future research could broaden the dataset to include videos from a wider range of platforms, including YouTube, Instagram, and Facebook, for a comparison of content across different platforms and regions. This would provide a more comprehensive evaluation of social media's role in global health education. Furthermore, including multilingual videos from different cultural contexts would enhance the generalizability of the findings and provide insights into the cross-cultural applicability of health communication strategies.

### Algorithmic and cross-cultural research

Further research could explore how platform algorithms influence the dissemination of health information across diverse cultures. This could involve studying the differences in algorithmic impact between Western and Eastern platforms and the role of algorithms in shaping health education across culturally diverse contexts.

### Academic contributions and practical implications

In summary, our study emphasizes the need for quality assessment of social media content, particularly on short-form video platforms. We identified significant disparities in the quality of OA-related videos across platforms and creators, underscoring the need for stricter content regulation and greater involvement of healthcare professionals in content creation. From a practical standpoint, enhancing the quality of health science communication on social media is essential. Short-form video platforms can play a pivotal role in chronic disease management by optimizing content presentation and advancing public health education, ultimately empowering individuals to take charge of their health.

## Conclusion

While short-video platforms serve as powerful tools for disseminating health information, substantial efforts are needed to enhance content quality. Ensuring the credibility, clarity, and actionability of health-related videos is crucial for optimizing their effectiveness in advancing public health education and empowering viewers with reliable, actionable knowledge.

## Data Availability

The original contributions presented in the study are included in the article/Supplementary Material, further inquiries can be directed to the corresponding author.
